# President Nunn’s Address before the Medical Association of Georgia

**Published:** 1886-06

**Authors:** 


### Editorial.

### PRESIDENT NUNN'S ADDRESS BEFORE THE MEDICAL ASSOCIATION OF GEORGIA.

We give a large amount of space this month to the President's address, delivered at the annual meeting of the Medical A sociation of Georgia. We feel that the importance of this address warrants its publication *in extenso*, since it treats of matters which should engage the earnest attention of every reader who has at heart the interests of the profession of this State. We propose to emphasize one or two points which seem to call for especial notice.

The strictures of Dr. Nunn upon the laws regulating the practice of medicine in this State are none too severe. We have long regarded these laws as a disgrace to our statute-books and to the law-making powers which placed them there. But having in mind the old maxim, *ex nihilo nihil fit*, how can it be expected that a body of men, who are so uncultured and illiterate that they cannot couch a law in grammatical and intelligent language, should be capable of grappling with the delicate questions involved in this subject. A legislative body which fails to appreciate the necessity of the study of practical anatomy as a preliminary to the practice of medicine, and which makes the pursuit of such study a penal offense, will naturally fail to discriminate between the itinerant charlatan and the scientific physician. When our legislators will abate their self-sufficiency to take counsel of the only class of men who are in position to give intelligent advice upon the subject, then, and not till then, will Georgia fall into line with Alabama, Illinois and other States in which the practice of medicine is *regulated* in fact as well as in name.

We desire especially to call attention to the portion of this address which treats of the pernicious and unethical practice of causing, or allowing to be inserted in secular newspapers, paragraphs or items which have for their object the advertisement of the names of individual physicians. This evil is recognized in unmistakable terms in the Code of Ethics of the American Medical Association and unequivocally condemned. But there are members of the profession in good standing in this, as well as in other cities, whose names appear so frequently in the columns of the daily press that it is patent to every intelligent reader that only by active suggestion or passive connivance can they occur. We feel warranted in taking the ground that the physician who accepts membership in the American Medical Association, or in any State or local association which adopts the Code, thereby binds himself in honor to conform to the requirements of that Code. To wilfully fail in this respect is to lay himself open to the charge of gross inconsistency, since he professes principles which he disregards in his daily practice. He is sailing under false colors. Shielding himself under the ægis of the Association, and accepting its benefits, emoluments and offices, he secretly violates its fundamental principles of which he is professedly an advocate. Such a man can boast of neither the frankness and independence of the so-called "New Code Men," nor the honesty and fidelity to principle of those who observe the letter and the spirit of the Code.

It is of course conceivable that a physician's name may accidentally appear in the public prints without his knowledge or consent. But it is a significant fact that such accidents occur repeatedly to certain men while others are never thus unfortunate. It cannot be said that a large practice with its resulting publicity exposes to such accidents, for they more frequently happen to the practitioner who feels the need of such adventitious aids. The busy man has no time to hobnob with newspaper reporters, nor does he feel the necessity of such a course. A resort to such methods may therefore be accepted as *prima facie* evidence of laxity of moral principle combined with a deficiency in the elements wh ch conduce to legitimate professional success.

Furthermore, laying aside the question of ethical propriety, and considering that of mere policy, it is doubtful if anything is ever gained by such practices. It is true that the "dear people" love to be deceived; but to successfully accomplish such deception requires the adroitness of an expert, and the men who surreptitiously advertise are rarely experts at anything. The public reads between the lines the palpable egotism and the undisguised desire for cheap notoriety, and then with a smile goes back to the old and tried family doctor whose name has never appeared in print during his half century of practice. It was wisely and truly said by Professor S. T. Clarke, of Buffalo, New York, on a recent occasion, that "the true place which you and I, as doctors, must find at last is the niche to which the profession assigns us." From this standpoint the physician who resorts to the newspaper puff will find in the long run that the niche which falls to his lot will be anything but an exalted one.

Our readers will discover other valuable suggestions in this able address. The two to which we have adverted seem to us especially timely and noticeable. We trust that the seed which is sown will bear fruit in due season.

